# Prediction of topological Dirac semimetal in Ca-based Zintl layered compounds CaM_2_X_2_ (M = Zn or Cd; X = N, P, As, Sb, or Bi)

**DOI:** 10.1038/s41598-022-08370-2

**Published:** 2022-03-17

**Authors:** Liang-Ying Feng, Rovi Angelo B. Villaos, Aniceto B. Maghirang, Zhi-Quan Huang, Chia-Hsiu Hsu, Hsin Lin, Feng-Chuan Chuang

**Affiliations:** 1 Department of Physics, National Sun Yat-Sen University, 70 Lienhai Rd., Kaohsiung, 80424 Taiwan; 2 Physics Division, National Center for Theoretical Sciences, Taipei, 10617 Taiwan; 3 Institute of Physics, Academia Sinica, Taipei, 115201 Taiwan; 4 Department of Physics, National Tsing Hua University, Hsinchu, 30013 Taiwan; 5 Center for Theoretical and Computational Physics, National Sun Yat-Sen University, Kaohsiung, 80424 Taiwan

**Keywords:** Topological insulators, Electronic properties and materials, Phase transitions and critical phenomena, Spintronics, Electronic structure

## Abstract

Topological Dirac materials are attracting a lot of attention because they offer exotic physical phenomena. An exhaustive search coupled with first-principles calculations was implemented to investigate 10 Zintl compounds with a chemical formula of CaM_2_X_2_ (M = Zn or Cd, X = N, P, As, Sb, or Bi) under three crystal structures: CaAl_2_Si_2_-, ThCr_2_Si_2_-, and BaCu_2_S_2_-type crystal phases. All of the materials were found to energetically prefer the CaAl_2_Si_2_-type structure based on total ground state energy calculations. Symmetry-based indicators are used to evaluate their topological properties. Interestingly, we found that CaM_2_Bi_2_ (M = Zn or Cd) are topological crystalline insulators. Further calculations under the hybrid functional approach and analysis using *k · p* model reveal that they exhibit topological Dirac semimetal (TDSM) states, where the four-fold degenerate Dirac points are located along the high symmetry line in-between Г to A points. These findings are verified through Green's function surface state calculations under HSE06. Finally, phonon spectra calculations revealed that CaCd_2_Bi_2_ is thermodynamically stable. The Zintl phase of AM_2_X_2_ compounds have not been identified in any topological material databases, thus can be a new playground in the search for new topological materials.

## Introduction

The investigations on topological materials (TMs) have been steadily gaining traction in the field of materials physics because they exhibit novel physical phenomena^1,2^. Intriguingly, the topological Dirac semimetal (TDSM) is an intermediate quantum state between trivial and non-trivial states^3^. Also, these new types of materials are a good playground to identify the new quantum phenomena in condensed matter physics from Dirac semimetals to Weyl semimetals^4,5^. TDSM is observed when the valence and conduction bands are degenerate at one critical point, defined as Dirac point in 3-dimension (3D) bulk structure near the Fermi level, and exhibits Lorentz symmetry breaking, which forms a Dirac cone^6,7^. Different types of cones at the Dirac point with or without inverse-sign slopes can be classified into either type I or type II Dirac semimetal, respectively^8^. TDSM has attracted significant attention because of its physical properties such as giant diamagnetism^9,10^, quantum magnetoresistance^11–14^, characteristic Landau level structures, and oscillating quantum spin Hall effect^15,16^. Recently, the TDSM state of materials has been experimentally observed in Cd_3_As_2_^14,17–20^ and Na_3_Bi^13,21^ as a type I Dirac semimetal. On the other hand, some of the transition metal dichalcogenides (TMDs) were proposed as a type II Dirac semimetal, such as PtSe_2_^22,23^, PdTe_2_^24,25^, PtTe_2_^26,27^, and NiTe_2_^28–31^, and were verified through angle-resolved photoemission spectroscopy (ARPES) experiments. In addition, numerous studies have demonstrated that compounds with hexagonal structure exhibit interesting electronic ^32,33,34^, and topological properties^35–39,40–42^.

Meanwhile, a group of materials called the Zintl compounds, named after Eduard Zintl who discovered them^43^, are receiving recognition in materials physics because of their applications in thermoelectric (TE) devices^44^. As more compounds were discovered, a more general description of these materials are now based on electron transfer between the alkali or alkaline earth cation and the electronegative elements which results in a filled valence shells through covalent bonding or formation of lone electron pairs. Therefore, these phases possess salt-like characteristics from the ionic bonding between the cation and the anion^43^. This definition of Zintl phase is a description of ionic and covalent bonding within intermetallic phases which provides insight on their structure and properties^45^. Recently, Zintl phases have been used as thermoelectric materials because of their structural complexity, low lattice thermal conductivity, and excellent stability at high temperature. Interestingly, some of the TE materials may exhibit topological properties due to their strong spin–orbit coupling (SOC) effect and small bandgap^46^. Interestingly, a new group of Zintl materials^47–51^ have been attracting attention because of their possible applications in the design and discovery of new quantum materials. Specifically, EuSn_2_P_2_^47^ and EuCd_2_As_2_^48^ have been recently experimentally and theoretically investigated. It was found that EuSn_2_P_2_ is antiferromagnetic and is an energetically stable state at low temperatures. Moreover, it was found to be an antiferromagnetic (AFM) topological insulator with Dirac-like surface states near the Fermi level. Similar to EuSn_2_P_2_, EuCd_2_As_2_ is also an antiferromagnet that exhibits three different types of non-trivial properties: axion insulator, AFM topological crystalline insulator (TCI), and higher order topological insulator. With these findings, EuCd_2_As_2_ is the first experimentally realized magnetic TDSM. In addition to these two materials, EuIn_2_As_2_^49^ and EuCd_2_Bi_2_^50^ have been theoretically predicted to exhibit magnetic topological insulator phase. These materials belong to a new group of exotic quantum materials known as axionic insulators^52,53^. With these exciting new findings in the Zintl compounds, it is crucial that an in-depth material search should be conducted to further explore this new playground of materials.

In this study, we investigate the 10 Zintl phase materials which have a general chemical formula of CaM_2_X_2_, where M = Cd or Zn, and X = N, P, As, Sb, or Bi. Through ground state energy and phonon dispersion calculations, we determined the most energetically favorable structure and found that all of these materials only adapt the hexagonal CaAl_2_Si_2_-type layered structure. Thus, we only focused on this layered-structure phase. Symmetry-based indicators are then used to evaluate their topological properties. Surprisingly, we found that CaM_2_Bi_2_ (M = Zn or Cd) are topological crystalline insulators (TCIs) under the GGA-PBE. Further calculations under the hybrid functional approach and analysis using *k · p* model reveal that they exhibit topological Dirac semimetal (TDSM) states, where the four-fold degenerate Dirac points are located along the high symmetry line in-between Г to A points. Our findings show that the Zintl phase of AM_2_X_2_ compounds can be a new playground in the search for new topological materials (TMs).

## Results and discussion

**Figure 1:**
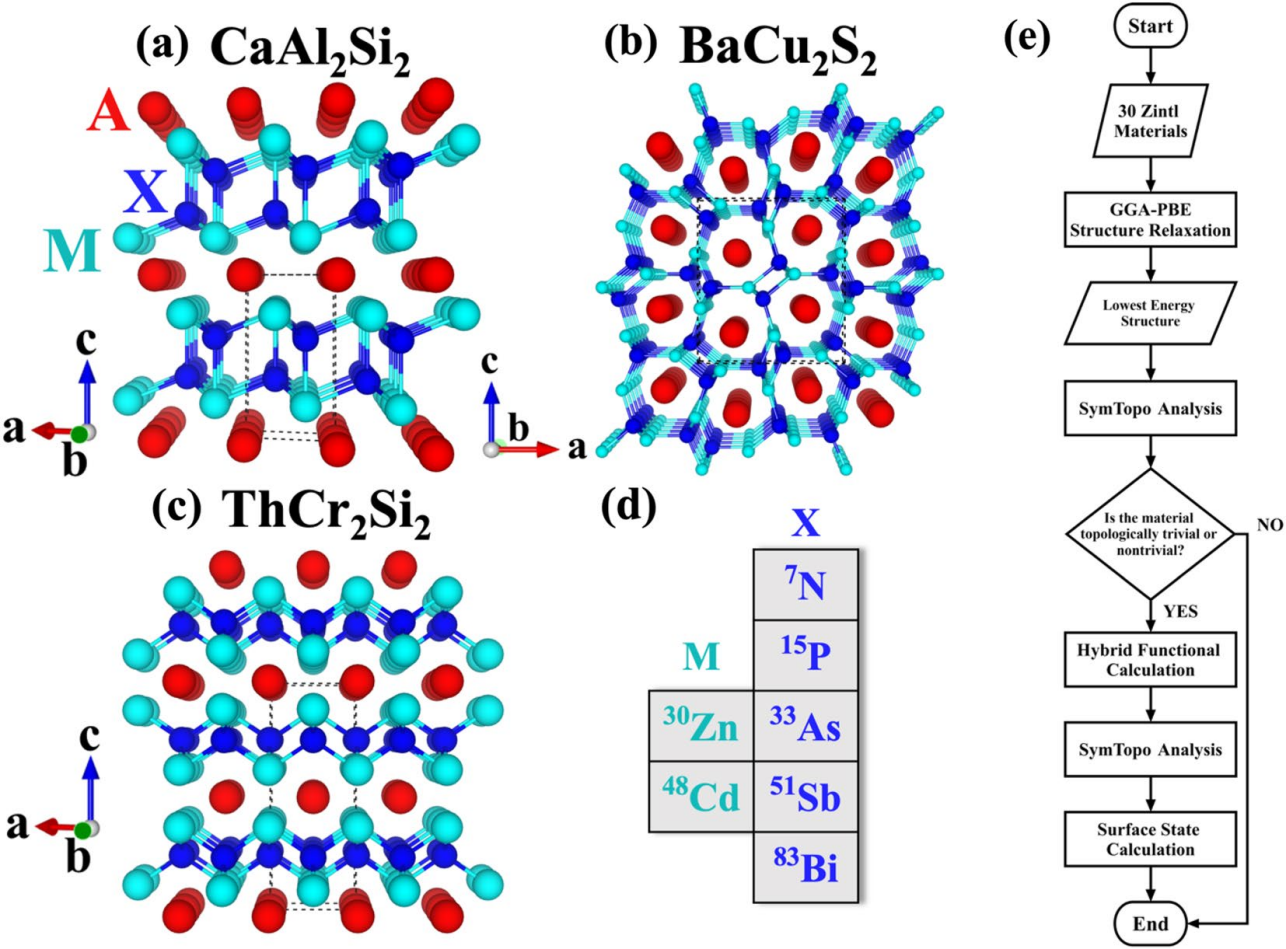
Three possible structures of bulk AM_2_X_2_: (**a**) CaAl_2_Si_2_-type (P$\overline{3 }$m1, 164) (**b**) BaCu_2_S_2_-type (Pnma, 62) (**c**) ThCr_2_Si_2_-type (I4/mmm, 139). (**d**) The list of elements used in this exhaustive materials search. M and X are, Group IIB, and Group VA, respectively. (**e**) The flow chart of our search for identifying the topological materials.

The AM_2_X_2_ Zintl compounds could exhibit three known structures: trigonal CaAl_2_Si_2_-type structure (P$\overline{3 }$m1, 164), orthorhombic BaCu_2_S_2_-type structure (Pnma, 62), and tetragonal ThCr_2_Si_2_-type structure (I4/mmm, 139), as shown in Fig. 1a–c, respectively^44^. The elements of CaM_2_X_2_ used in our study are shown in Fig. 1d. The M are Zn or Cd from Group 2B (Zinc Group), and X are N, P, As, Sb, or Bi from Group 5A (Pnictogen Group). The CaAl_2_Si_2_-type structure is stacked along the *c-*direction by two-layer structures A^2+^ and [M_2_X_2_]^2-^. On the other hand, the orthorhombic BaCu_2_S_2_-type structure is formed through the covalent bonding between M and X. This results in a channel-like structure along the lattice vector *b* with A atoms inside these channels as shown in Fig. 1b. Finally, the ThCr_2_Si_2_-type structure is composed of negatively charged layers of MX_4_ tetrahedra and positively charged A layers, stacked along the *c* direction. The MX_4_ tetrahedra layers contain strong covalent M–X bonds and weaker M–M interactions, while an ionic bonding exists between A and the MX_4_ tetrahedra layers^54^.

To systematically study the 10 Zintl compounds, the exhaustive search approach is adapted. The procedures are depicted in the flowchart shown in Fig. 1e. First, we assigned the 10 compounds to the three known structures in the literature, totaling to 30 materials, and determined the most stable structure of each compound using the GGA-PBE functional. Second, the topological properties of the 10 compounds with the lowest ground state energy structure were determined using the SymTopo program^55^ via the symmetry-indicator analysis. The band structures were also calculated to determine their electronic properties: insulating, semi-metallic, or metallic. Third, hybrid functional calculations combined with the SymTopo program^55^ were used again to verify their topological and electronic properties. Finally, the evidence of topological properties is further explored through the surface Green’s function calculations.

The results of the exhaustive search procedure, as well as the topological phases of each material are summarized in Supplementary Table S1 (without SOC) and S2 (with SOC). For the 10 compounds considered, they all adapt the CaAl_2_Si_2_-type structure. Nevertheless, the bulk band structures for the BaCu_2_S_2_-type and ThCr_2_Si_2_-type CaM_2_X_2_ compounds considered in this study are shown in Supplementary Figures S1 to S4. The SymTopo analysis indicated that two compounds, CaZn_2_Bi_2_ and CaCd_2_Bi_2_, are found to be TCSMs, and CaCd_2_N_2_ would be TSM while the rest are trivial topological properties under GGA. Moreover, phonon dispersion calculations were performed to verify the thermodynamic stability of the CaAl_2_Si_2_-type CaM_2_X_2_ compounds (See Figure S5). As seen in Figure S5, majority of the bulk materials, except for CaZn_2_Bi_2_, considered in this study have no imaginary phonon frequencies indicating thermodynamic stability. On the other hand, CaZn_2_Bi_2_ in CaAl_2_Si_2_-type exhibits imaginary phonon frequencies and is thermodynamically unstable, implying it may adopt another stable structure. Interestingly, the monolayer CaZn_2_Bi_2_ is stable, as demonstrated in another study^56^. Focusing on CaCd_2_Bi_2_, Figure S5 shows that CaCd_2_Bi_2_ is thermodynamically stable, implying possible experimental synthesis. Also, the phase diagrams for CaCd_2_Sb_2_ and CaCd_2_Bi_2_ from the Open Quantum Materials Database^57,58^ show that both materials have negative formation energies, implying relative stability. Furthermore, experimental realization of this material is indeed possible since CaCd_2_Sb_2_ with similar crystal structure have already been synthesized^59^. Intriguingly, these materials have not been identified in several topological material databases^60–62^. The band structures of these 10 compounds are shown in Supplementary Figure S6 and S7. To verify the results, all the compounds are recalculated again with HSE06 as shown in Figures S8 and S9. Interestingly, CaCd_2_Bi_2_ and CaZn_2_Bi_2_ are found to be TDSM, while the other 8 materials just have trivial insulating phase. To demonstrate the topological properties of these two materials, we choose CaCd_2_Bi_2_ as the representative material for the rest of our discussion.

As seen in Supplementary Table S1 and Figures S6 (a, c, e, g, and i) and S7 (a, c, e, g, and i) under GGA-PBE without SOC, the SymTopo results and band structures, respectively, reveal that CaCd_2_Sb_2_ is a trivial insulator (Figure S7g) while CaCd_2_Bi_2_ is a topological semimetal (TSM) (Figure S7i). When turning on SOC, CaCd_2_Sb_2_ is a trivial semimetal (SM) (Figure S7h), while CaCd_2_Bi_2_ is a topological crystalline semimetal (TCSM) (Figure S7j). Here, the observed transition from trivial SM to TCSM in CaCd_2_Bi_2_ is due to the SOC effect, meaning the bands are gapped between Γ to A. In order to understand the evolution of topological phase transitions, the SOC strength of CaCd_2_Bi_2_ is varied from 0 to 1. The bands gradually open at two symmetry points, one is at Γ while the other is at A point, as the SOC strength increases. This band opening explains the mechanism behind the TCSM property of CaCd_2_Bi_2_ (see Supplementary Figures S10 and S11). This similar behavior is also observed in Sb/Bi planar honeycomb which exhibits topological crystalline insulator phase^63^.

**Figure 2:**
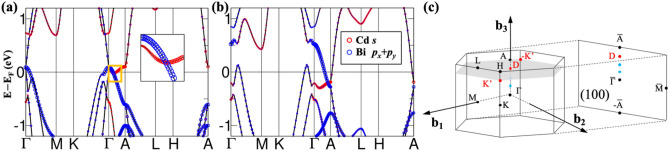
The band structures of bulk CaCd_2_Bi_2_ under HSE06 (**a**) w/o SOC and (**b**) with SOC. The circle corresponds to the orbital contribution from Cd *s* orbital and Bi *p*_*x*_ and *p*_*y*_ orbitals indicated by red and blue, respectively. To identify the bands near Γ point of (**a**), the zoom-in diagram was provided in the inset. (**c**) The corresponding 3D first Brillouin zone (BZ) of the CaAl_2_Si_2_-type structure and the projected surface BZ at (100) plane. The high-symmetry points and Dirac points (red for CaCd_2_Bi_2_ and blue for CaCd_2_SbBi) are labeled in the figure.

Moving on to the results under the hybrid functional approach, we show in Fig. 2a, b the band structures of CaCd_2_Bi_2_ under HSE06 with and without SOC. Since HSE06 has been proven to be more superior than the standard GGA-PBE functional, we will focus only on HSE06 results for the succeeding discussions unless mentioned otherwise. Interestingly, CaCd_2_Bi_2_ is a TSM without SOC (Fig. 2a) exhibited the characteristics of a nodal line semimetal with degenerate state (bands are contributed by Bi *p*_*x*_ and *p*_*y*_ orbitals) along Γ to A as highlighted by the yellow square, but became a TDSM under SOC (Fig. 2b) with a Dirac point crossing observed along Γ to A. The locations of Dirac points are in red labeled in Fig. 2c.

**Figure 3:**
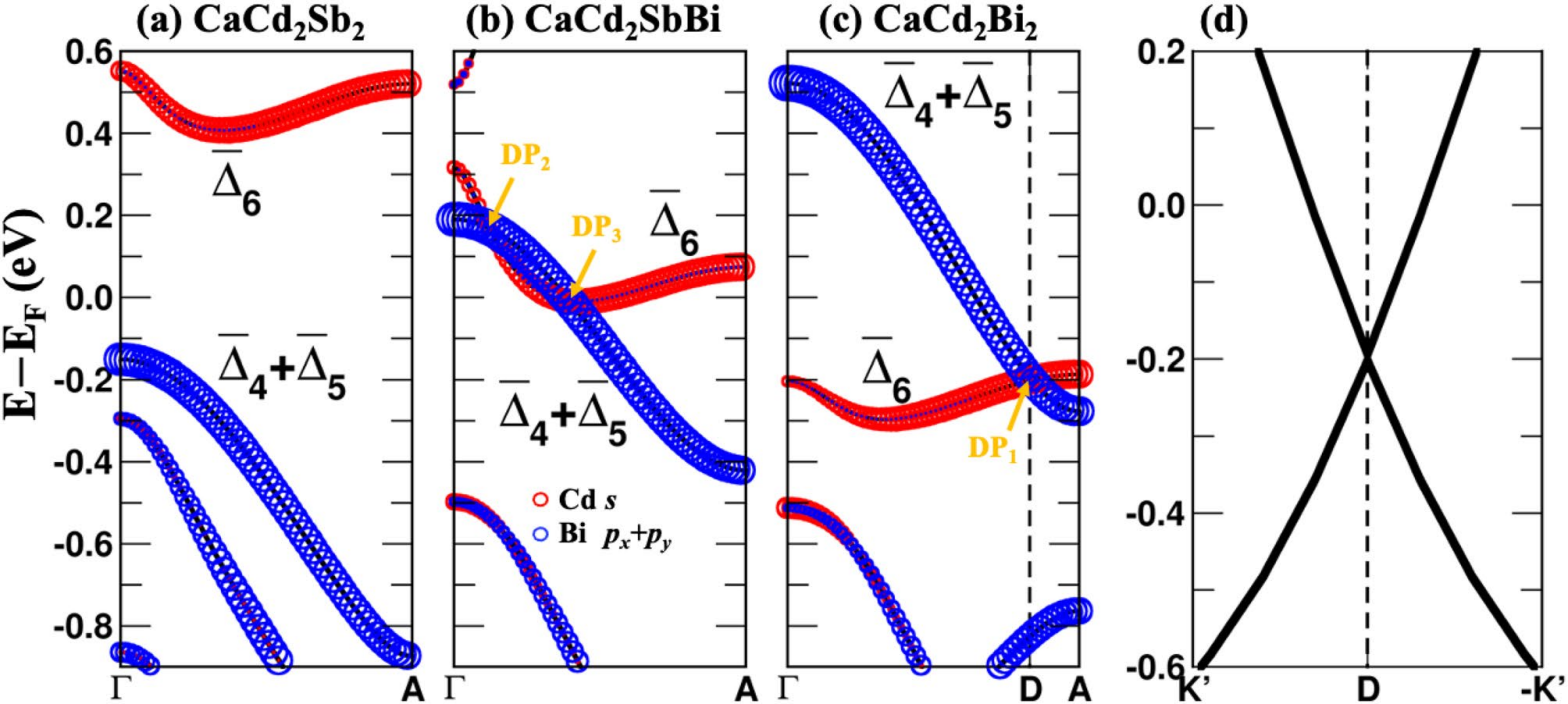
(**a**–**c**) The HSE06 with SOC band structures of CaCd_2_Sb_2_, CaCd_2_SbBi, and CaCd_2_Bi_2_. The circle corresponds to the orbital contribution from Cd *s* orbital and Bi *p*_*x*_ and *p*_*y*_ orbitals indicated by red and blue, respectively. The irreducible representations are labeled as ${\overline{\Delta } }_{6}$ and ${\overline{\Delta } }_{4}+{\overline{\Delta } }_{5}$. The three Dirac points are labeled in orange arrows (DP_1_, DP_2_, and DP_3_). (**d**) Zoomed-in view of the band path near passing the Dirac cone of CaCd_2_Bi_2_ along the -K’ to D to K’.

To understand the mechanism behind the existence of the Dirac point, the zoomed-in with SOC band structures of CaCd_2_Sb_2_ and CaCd_2_Bi_2_ along Γ to A are shown in Fig. 3a, c. For CaCd_2_Sb_2_, there exists a band opening with a gap of 0.55 eV resulting in a trivial insulator phase. In contrast, CaCd_2_Bi_2_ demonstrates gapless linear bands crossing in (0, 0, k_D_ ≈ ± 0.4152π/c) at the energy level of E − E_F_ ≈ −0.20 eV. It is a four-fold degenerate Dirac point (DP_1_) belonging to two different irreducible representations (${\overline{\Delta } }_{4}+{\overline{\Delta } }_{5}$ and ${\overline{\Delta } }_{6}$) which prohibits hybridization, as shown in Fig. 3c. To prove that it is indeed a 3D Dirac point, we show in Fig. 3d the band path passing the Dirac cone of CaCd_2_Bi_2_ along the DP_1_ to -K’ and DP_1_ to K’, which is perpendicular to Γ to A direction. This result demonstrated the existence of a TDSM state in CaCd_2_Bi_2_. The Dirac point (DP_1_) is protected by the C_3_ rotational symmetry about the *c* axis.

To explore the topological phase transition due to SOC strength and doping, we propose a hypothetical material CaCd_2_SbBi that has an equal concentration of Sb and Bi. In CaCd_2_SbBi, Bi doping breaks the inversion symmetry of the system. The CaCd_2_SbBi in CaAl_2_Si_2_-type structure belongs to the space group P$\overline{3 }$m1 (164), while the doped case belongs to P3m1 (156). For comparison, Fig. 3b also shows the band structure of CaCd_2_SbBi along the Γ to A under HSE06. Interestingly, the substituted Bi in CaCd_2_SbBi causes the breaking of the inversion symmetry while preserving the C_3_ rotational symmetry along the z-axis. From Fig. 3a–c, we observed the down shift of ${\overline{\Delta } }_{6}$ and upward shift of ${\overline{\Delta } }_{4}+{\overline{\Delta } }_{5}$. Two bands, ${\overline{\Delta } }_{4}+{\overline{\Delta } }_{5}$ and ${\overline{\Delta } }_{6}$, near the Fermi level closed and crossed over the Fermi level resulting in a pair of Dirac points (DP_2_ and DP_3_) in the first BZ, as shown by a pair of the blue points labeled in Fig. 2c. Surprisingly, the symmetry indicators at Γ and A are still the same as CaCd_2_Sb_2_, thus resulting in a trivial insulator phase. Totally, four emerging Dirac points are formed along -A to Γ to A. Eventually, one Dirac point remains as the material is changed to CaCd_2_Bi_2_.

To further elucidate the mechanism behind the TDSM in CaCd_2_Bi_2_, the band crossing between Γ and A points is expressed as an effective Hamiltonian. To prove that the fourfold point is a Dirac point, an analysis using the *k · p* model is discussed. The four-band effective Hamiltonian obeys the following three symmetries: threefold rotation symmetry $({C}_{3}^{+})$, mirror symmetry (*σ*_*1v*_), and inversion and time reversal symmetry (*IT*)^64^. The irreducible representations of the four-band at D (Fig. 3c, d), ${\overline{\Delta } }_{4}+{\overline{\Delta } }_{5}$ and ${\overline{\Delta } }_{6}$, are chosen as the basis set. Using the Pauli matrices *σ*_*1*_*, σ*_*2*_*, σ*_*3*_, and 2 × 2 identity matrix *σ*_*0*_, the matrix representations of the three symmetries for the basis are − *σ*_*0*_*, iσ*_*3*_*, − iσ*_*2*_ and $\frac{1}{2}$*(σ*_*0*_* − i*$\sqrt{3}$*σ*_*2*_*), iσ*_*3*_*, − iσ*_*2*_, respectively. Thus, the four-band effective Hamiltonian takes the form

$$\left[\begin{array}{cccc}{c}_{1}+{c}_{2}{k}_{z}+{c}_{3}{k}_{z}& 0& {c}_{4}{k}_{y}-i{c}_{5}{k}_{y}& {c}_{4}{k}_{x}-i{c}_{5}{k}_{x}\\ 0& {c}_{1}+{c}_{2}{k}_{z}+{c}_{3}{k}_{z}& {c}_{4}{k}_{x}+i{c}_{5}{k}_{x}& {-c}_{4}{k}_{y}-i{c}_{5}{k}_{y}\\ {c}_{4}{k}_{y}+i{c}_{5}{k}_{y}& {c}_{4}{k}_{x}-i{c}_{5}{k}_{x}& {c}_{1}+{c}_{2}{k}_{z}-{c}_{3}{k}_{z}& 0\\ {c}_{4}{k}_{x}+i{c}_{5}{k}_{x}& {-c}_{4}{k}_{y}+i{c}_{5}{k}_{y}& 0& {c}_{1}+{c}_{2}{k}_{z}-{c}_{3}{k}_{z}\end{array}\right]$$

The eigenvalues of the four-bands *(c*_*1*_ + *c*_*2*_*k*_*z*_*)*±$\sqrt{({c}_{4}^{2}+{c}_{5}^{2}){k}_{x}^{2}+({c}_{4}^{2}+{c}_{5}^{2}){k}_{y}^{2}+{c}_{3}^{2}{k}_{z}^{2}}$ indicates that the four-fold point is indeed a Dirac point^64^.

**Figure 4:**
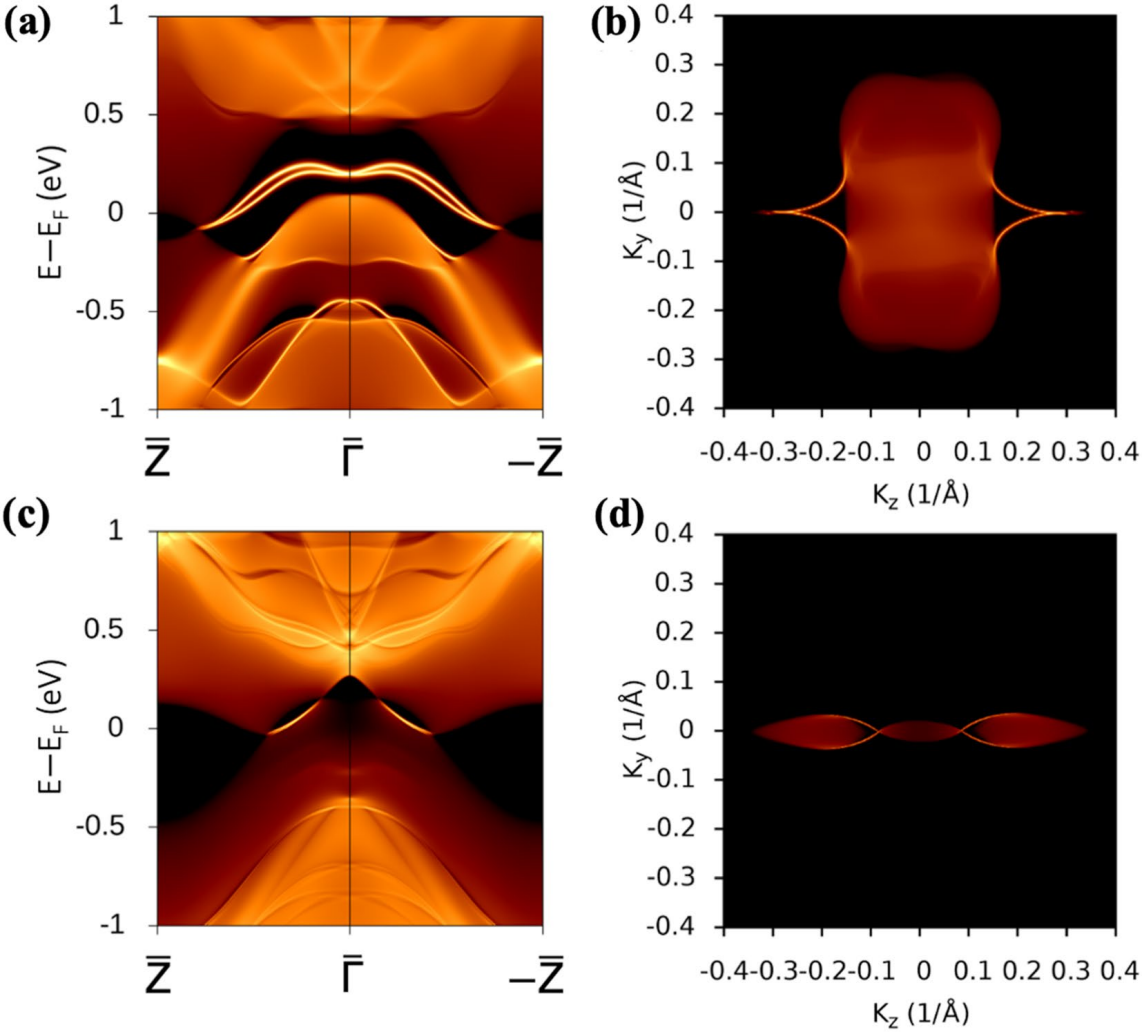
Bulk (**a**) CaCd_2_Bi_2_ and (**c**) CaCd_2_SbBi surface states projected on (100) plane under HSE06 with SOC. 2D Fermi arcs for (**b**) CaCd_2_Bi_2_ and (**d**) CaCd_2_SbBi at the E−E_F_ = −0.1 eV and 0.1 eV, respectively.

Finally, the Green’s function-derived surface states projected on the (100) plane are calculated for CaCd_2_Bi_2_ (Fig. 4a) and CaCd_2_SbBi (Fig. 4c) to confirm the location of Dirac points. The 3D band structure is shown in Figure S12 highlighting two Dirac cones. The corresponding Fermi arcs highlighting the surface states of CaCd_2_Bi_2_ and CaCd_2_SbBi are shown in Fig. 4b, d, respectively. The connections to the Dirac points are projected onto different *k* locations in the surface BZ. The *k* points in the BZ projected on (100) surface are shown in Fig. 2c. The (100) surface of the Γ-centered BZ corresponds to the projection of the M-Γ line. In Fig. 4a, the calculated surface states connect two Dirac points (DP_1_) in CaCd_2_Bi_2_ and cross at Γ point. However, since the structure of CaCd_2_SbBi breaks the inversion symmetry, four emerging Dirac points are formed. In Fig. 4c, the surface states of CaCd_2_SbBi, in contrast with CaCd_2_Bi_2_, do not cross at a high symmetry point while connecting each pair of Dirac points (DP_2_ and DP_3_) in the BZ. The surface states connect the two Dirac points (DP_2_ and DP_3_) within Γ to A, while the other pair of surface states connect the two Dirac points within Γ to −A.

## Methods

We systematically explored 10 compounds of CaM_2_X_2_ where M = Zn or Cd; and X = N, P, As, Sb, or Bi, under the three different existing structures, totaling 30 compounds of Zintl materials: CaAl_2_Si_2_-, ThCr_2_Si_2_-, and BaCu_2_S_2_-type crystal phase^44^, through first-principles calculation as implemented in Vienna Ab initio Simulation Package (VASP) using projector-augmented wave (PAW) functions^65,66^ under the Perdew-Burke-Ernzerhof (PBE)^67^ functional with plane wave cut-off energy of 400 eV. The structures were allowed to relax until the residual Hellmann–Feynman forces acting on each atom were no greater than 10^–2^ eV/Å. The K-points sampling used is the Γ-centered grid Monkhorst–Pack with 24 × 24 × 12 kmesh within the first Brillouin zone (BZ) for all the self-consistent calculations. Phonon spectra calculations are performed using the Phonopy^68^ package. Spin-polarized and SOC were included in all the energetic calculations. In determining the magnetic properties, we treat magnetic moments (μ) greater than 0.1 as ferromagnetic (FM) states, while the rest are non-magnetic (NM) states. In NM state, we analyzed each case for the topological properties in 3D bulk structure using the SymTopo package^55^ which uses symmetry-based indicators that can categorize different topological properties, such as trivial insulator (I), conventional metal (M), topological insulator (TI), topological crystalline insulator (TCI), high-symmetry-point semi-metal (HSPSM), and high-symmetry-line semi-metal (HSLSM). To predict the topological properties of the investigate materials more accurately, Heyd-Scuseria-Ernzerhof (HSE06) hybrid functional^69^ is further included in the band structure calculations. Finally, the Maximally-Localised Wannier Functions (MLWF) of CaM_2_Bi_2_ are obtained from the Wannier90^70^ program, and the surface Green’s function is calculated using the WannierTools program^71^.

## Conclusions

In this study, a systematic materials search coupled with first-principles calculations was performed to investigate 10 Zintl-phase compounds with a chemical formula of CaM_2_X_2_ (M = Zn or Cd; X = N, P, As, Sb, or Bi) and found all of these materials adapt CaAl_2_Si_2_-type structure. Symmetry-based indicators are used to evaluate their topological properties. Surprisingly, we found that CaM_2_Bi_2_ (M = Zn or Cd) are topological crystalline insulators (TCIs) under the GGA-PBE. Further calculations under the hybrid functional approach reveal that they exhibit topological Dirac semimetal (TDSM) states, where the four-fold degenerate Dirac points are located along the high symmetry line in-between Г to A points and is analyzed using the effective Hamiltonian derived by the *k · p* model. These findings are verified through Green's function surface state calculations under HSE06. Finally, phonon calculations were done to verify the thermodynamic stability of the CaCd_2_Bi_2_, implying possible synthesis. Our findings show that the Zintl phase of AM_2_X_2_ compounds can be a new playground in the search for new topological materials (TMs).

## Supplementary Information

Supplementary Information.
