# Supplementary material for: Prediction of topological Dirac semimetal in Ca-based Zintl layered compounds CaM2X2 (M = Zn or Cd; X = N, P, As, Sb, or Bi)
Source: Sci Rep. 2022 Mar 17;12:4582. doi: 10.1038/s41598-022-08370-2 (PMC8930984; doi:10.1038/s41598-022-08370-2)
Supplement: Supplementary file 1 — Supplementary Information. [file 41598_2022_8370_MOESM1_ESM.pdf]

Supplementary Information for "Prediction of topological Dirac semimetal in Ca - based Zintl layered compounds CaM_2X_2 (M = Zn or Cd; X = N, P, As, Sb, or Bi)"

Liang-Ying Feng[1], Rovi Angelo B. Villaos[1], Aniceto B. Maghirang III[1], Zhi-Quan Huang[1], Chia-Hsiu Hsu[1,2], Hsin Lin[3], and Feng-Chuan Chuang[1,2,4,5,6,]*

[1]Department of Physics, National Sun Yat-sen University, Kaohsiung, 80424 Taiwan

[2]Physics Division, National Center for Theoretical Sciences, Taipei, 10617 Taiwan

[3]Institute of Physics, Academia Sinica, Taipei, 115201 Taiwan

[4]Department of Physics, National Tsing Hua University, Hsinchu, 30013 Taiwan

[5]Center for Theoretical and Computational Physics, National Sun Yat-sen University, Kaohsiung 80424, Taiwan

[6]Center of Crystal Research, National Sun Yat-sen University, Kaohsiung 80424, Taiwan

[*] Corresponding Author

Postal Address: 70 Lienhai Rd., Kaohsiung 80424, Taiwan.

E-mail Address: fchuang@mail.nsysu.edu.tw

Phone number: +886 752523733

Table S1. List of topological properties of bulk CaM_2X_2 (M = Zn or Cd; X = N, P, As, Sb, or Bi) compounds under GGA-PBE without SOC. The I, M, SM, and TSM are abbreviations for trivial insulator, metal, trivial semimetal, and topological semimetal, respectively.

	**$CaAl_2Si_2$-type**	**$BaCu_2S_2$-type**	**$ThCr_2Si_2$-type**
**$CaZn_2N_2$**	I	I	I
**$CaZn_2P_2$**	I	I	I
**$CaZn_2As_2$**	I	I	SM
**$CaZn_2Sb_2$**	M	I	SM
**$CaZn_2Bi_2$**	TSM	TSM	TSM
**$CaCd_2N_2$**	TSM	TSM	TSM
**$CaCd_2P_2$**	I	I	TSM
**$CaCd_2As_2$**	I	TSM	TSM
**$CaCd_2Sb_2$**	I	I	TSM
**$CaCd_2Bi_2$**	TSM	TSM	TSM

Table S2. List of topological properties of bulk CaM_2X_2 (M = Zn or Cd; X = N, P, As, Sb, or Bi) compounds under GGA-PBE with SOC. The I, SM, TI, TSM, and TCSM are abbreviations for trivial insulator, trivial semimetal, topological insulator, topological semimetal, and topological crystalline semimetal, respectively.

	**$CaAl_2Si_2$-type**	**$BaCu_2S_2$-type**	**$ThCr_2Si_2$-type**
**$CaZn_2N_2$**	I	I	I
**$CaZn_2P_2$**	I	I	I
**$CaZn_2As_2$**	I	I	SM
**$CaZn_2Sb_2$**	I	SM	SM
**$CaZn_2Bi_2$**	TCSM	SM	TI
**$CaCd_2N_2$**	TSM	TI	TI
**$CaCd_2P_2$**	I	I	TSM
**$CaCd_2As_2$**	I	TI	TSM
**$CaCd_2Sb_2$**	SM	SM	TSM
**$CaCd_2Bi_2$**	TCSM	TSM	TSM

Table S3. The total ground state energy of CaM_2X_2 (M = Zn or Cd; X = N, P, As, Sb, or Bi) in electron volt per formula unit (eV/f.u.) under GGA-PBE without and with SOC for the three possible bulk CaM_2X_2 structures. The red colored energy values indicate the most stable structure.

	$CaAl_2Si_2$-type		$BaCu_2S_2$-type		$ThCr_2Si_2$-type	
	w/o SOC	with SOC	w/o SOC	with SOC	w/o SOC	with SOC
$CaZn_2N_2$	-22.6848	**-22.6964**	-22.2177	-22.2293	-21.2433	-21.2551
$CaZn_2P_2$	-18.4534	**-18.4658**	-18.0110	-18.0237	-17.6135	-17.6262
$CaZn_2As_2$	-16.7749	**-16.8100**	-16.3440	-16.3796	-16.0889	-16.1246
$CaZn_2Sb_2$	-14.6653	**-14.7971**	-14.3795	-14.5123	-14.0584	-14.1925
$CaZn_2Bi_2$	-13.3142	**-14.8337**	-13.2215	-14.7376	-12.9448	-14.4823
$CaCd_2N_2$	-20.1567	**-20.2181**	-19.7676	-19.8286	-17.8715	-17.9350
$CaCd_2P_2$	-17.0747	**-17.1381**	-16.3115	-16.3758	-15.8655	-15.9300
$CaCd_2As_2$	-15.7360	**-15.8233**	-14.9841	-15.0732	-14.8114	-14.9012
$CaCd_2Sb_2$	-14.0228	**-14.2092**	-13.4375	-13.6278	-13.2653	-13.4611
$CaCd_2Bi_2$	-12.8804	**-14.4811**	-12.5065	-14.1534	-12.4194	-14.0581

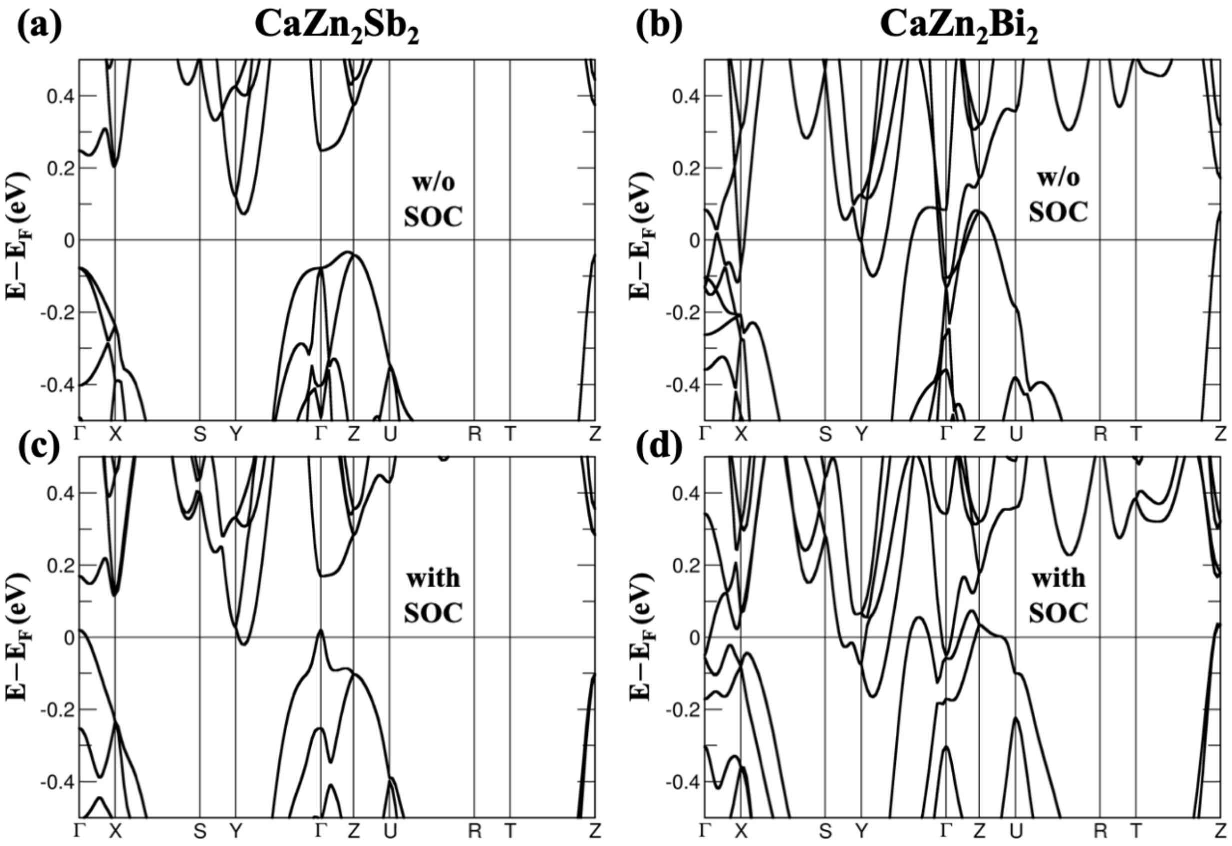

Figure S1. The band structures of bulk $CaZn_2X_2$ (X = Sb and Bi) under GGA-PBE (a-b) without SOC and (c-d) with SOC in the $BaCu_2S_2$-type structure.

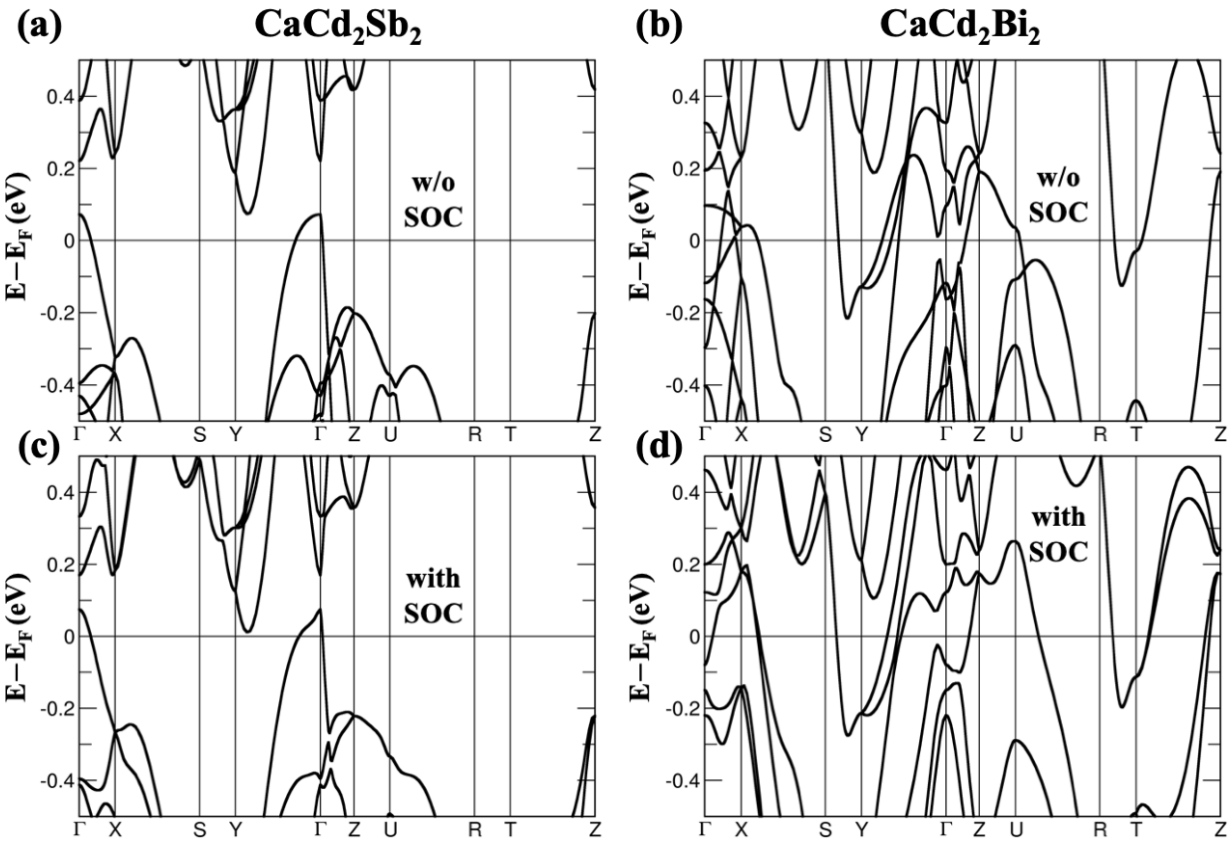

Figure S2. The band structures of bulk $CaCd_2X_2$ (X = Sb and Bi) under GGA-PBE (a-b) without SOC and (c-d) with SOC in the $BaCu_2S_2$-type structure.

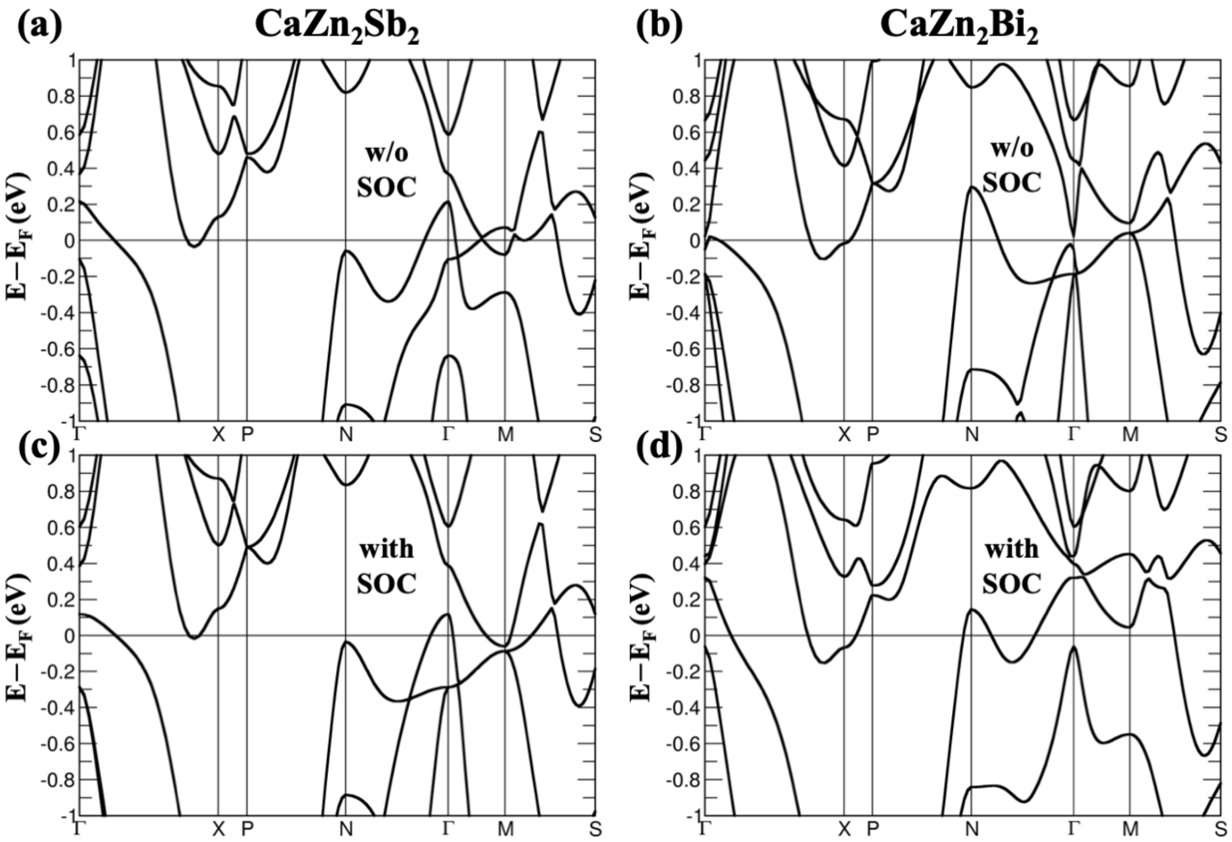

Figure S3. The band structures of bulk $CaZn_2X_2$ (X = Sb and Bi) under GGA-PBE (a-b) without SOC and (c-d) with SOC in the $ThCr_2Si_2$-type structure.

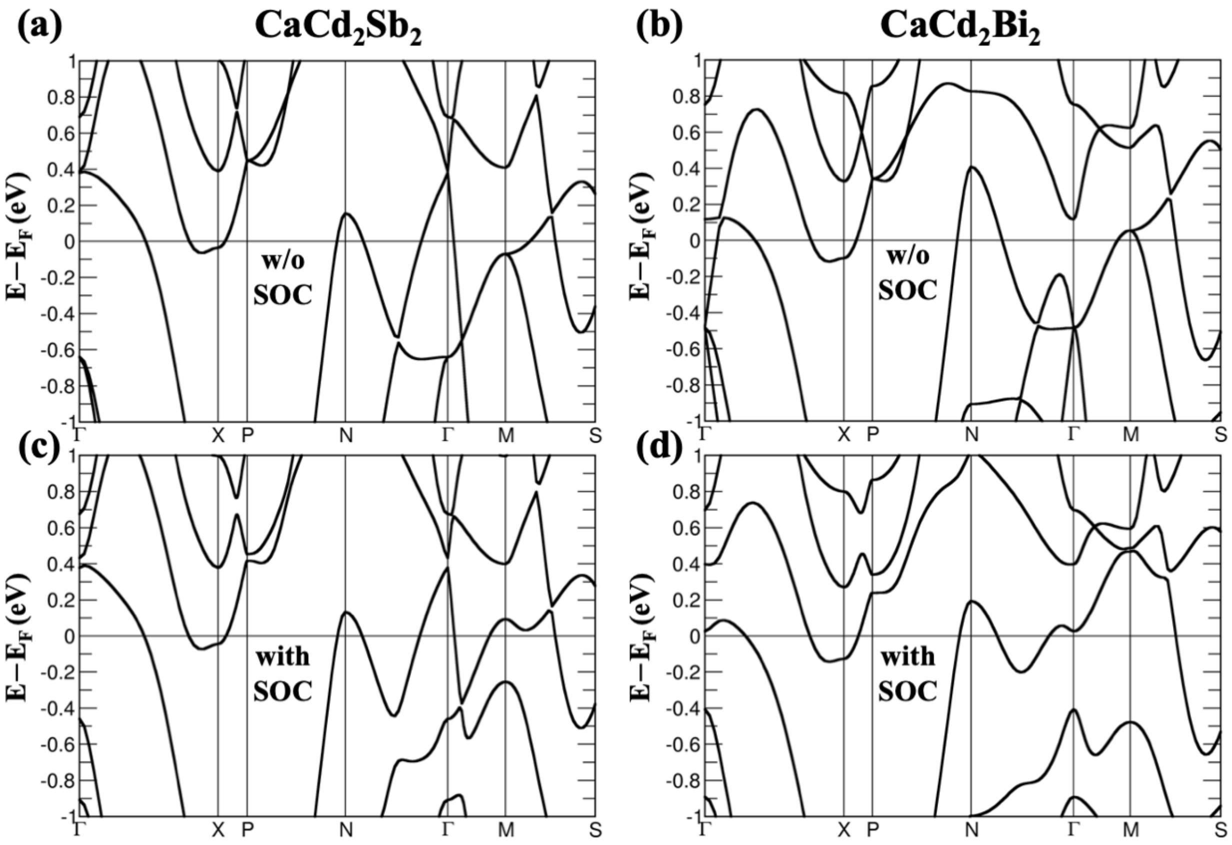

Figure S4. The band structures of bulk $CaCd_2X_2$ (X = Sb and Bi) under GGA-PBE (a-b) with SOC and (c-d) without SOC in the $ThCr_2Si_2$-type structure.

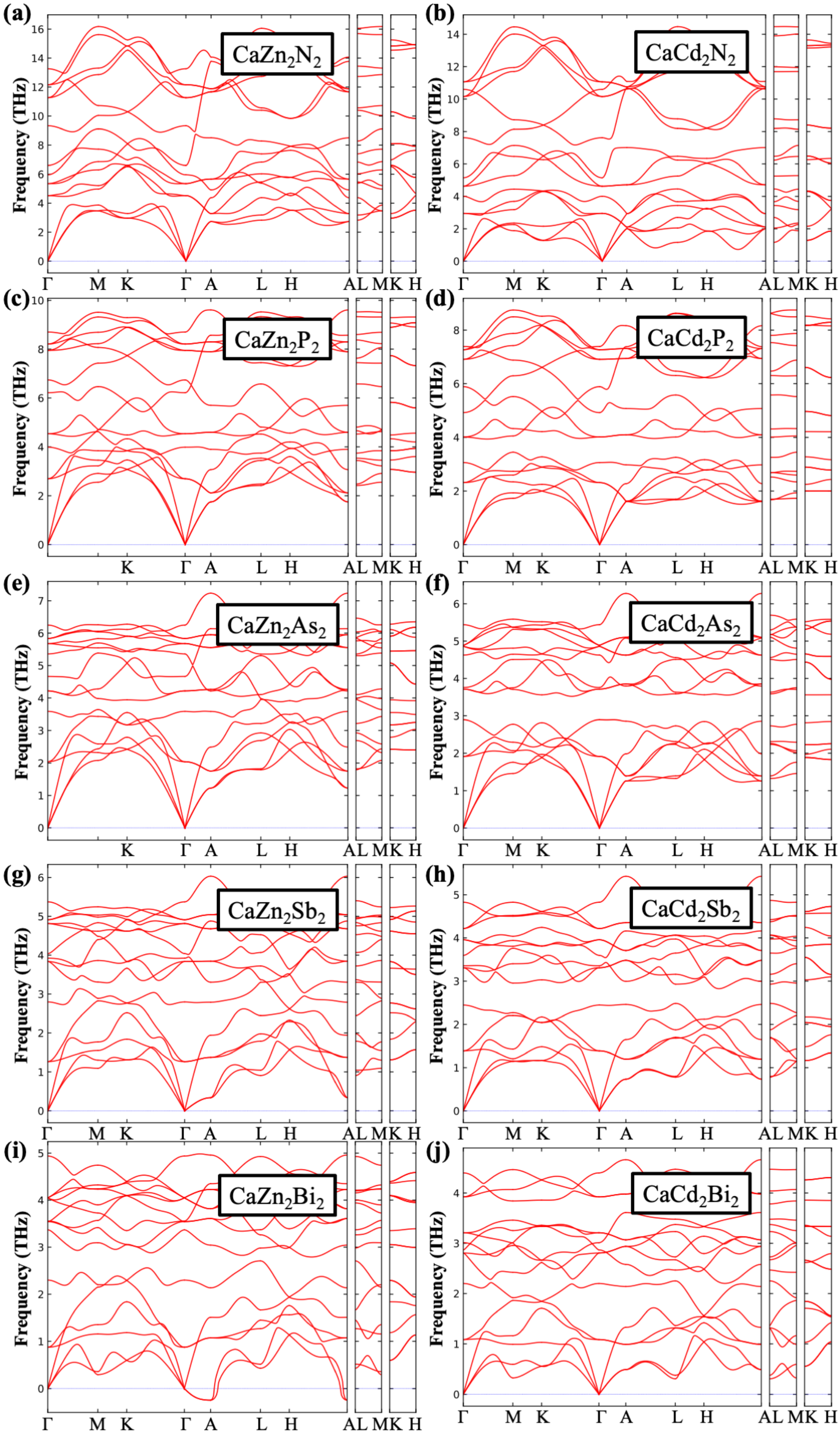

Figure S5. Phonon dispersions of bulk (a, c, e, g, and i) $CaZn_2X_2$ and (b, d, f, h, and j) $CaCd_2X_2$ (X = N, P, As, Sb, or Bi) in the $CaAl_2Si_2$-type structure.

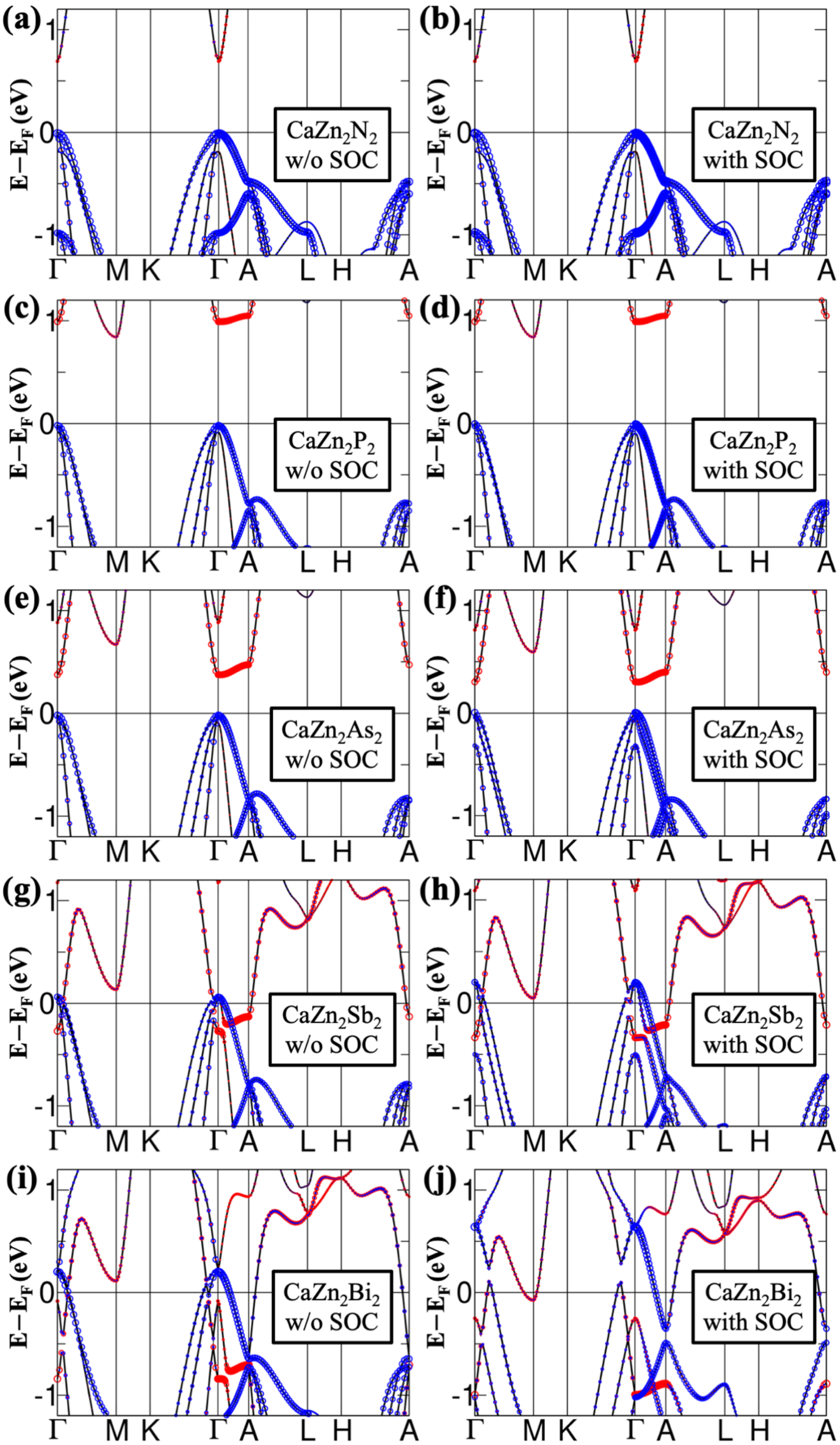

Figure S6. The band structures of bulk $CaZn_2X_2$ (X = N, P, As, Sb, or Bi) under GGA-PBE (a, c, e, g, and i) without SOC and (b, d, f, h, and j) with SOC in the $CaAl_2Si_2$-type structure. The circle corresponds to the orbital contribution from Cd *s* orbital and Bi p_x and p_y orbitals indicated by red and blue, respectively.

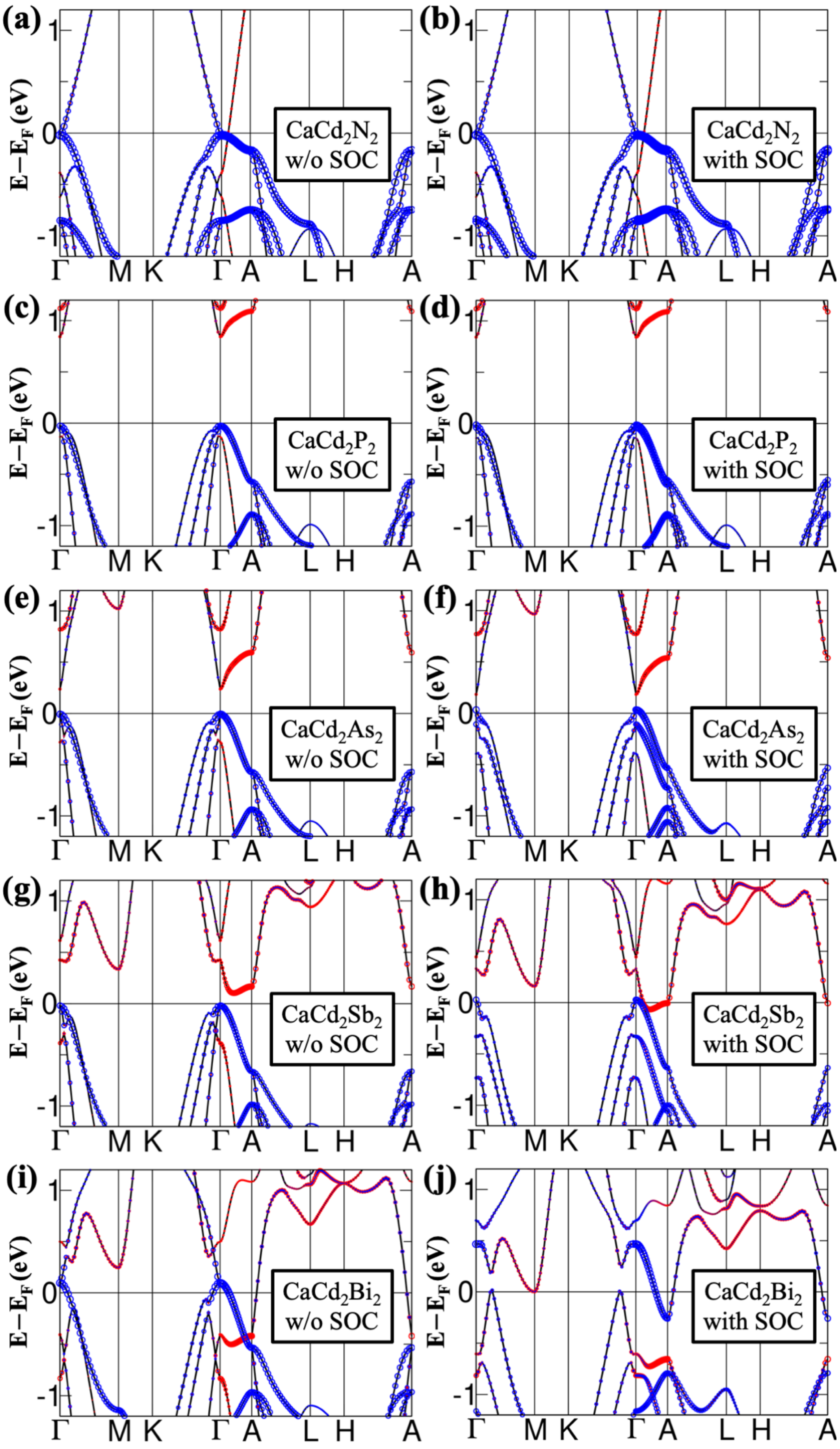

Figure S7. The band structures of bulk $CaCd_2X_2$ (X = N, P, As, Sb, or Bi) under GGA-PBE (a, c, e, g, and i) without SOC and (b, d, f, h, and j) with SOC in the $CaAl_2Si_2$-type structure. The circle corresponds to the orbital contribution from Cd *s* orbital and Bi p_x and p_y orbitals indicated by red and blue, respectively.

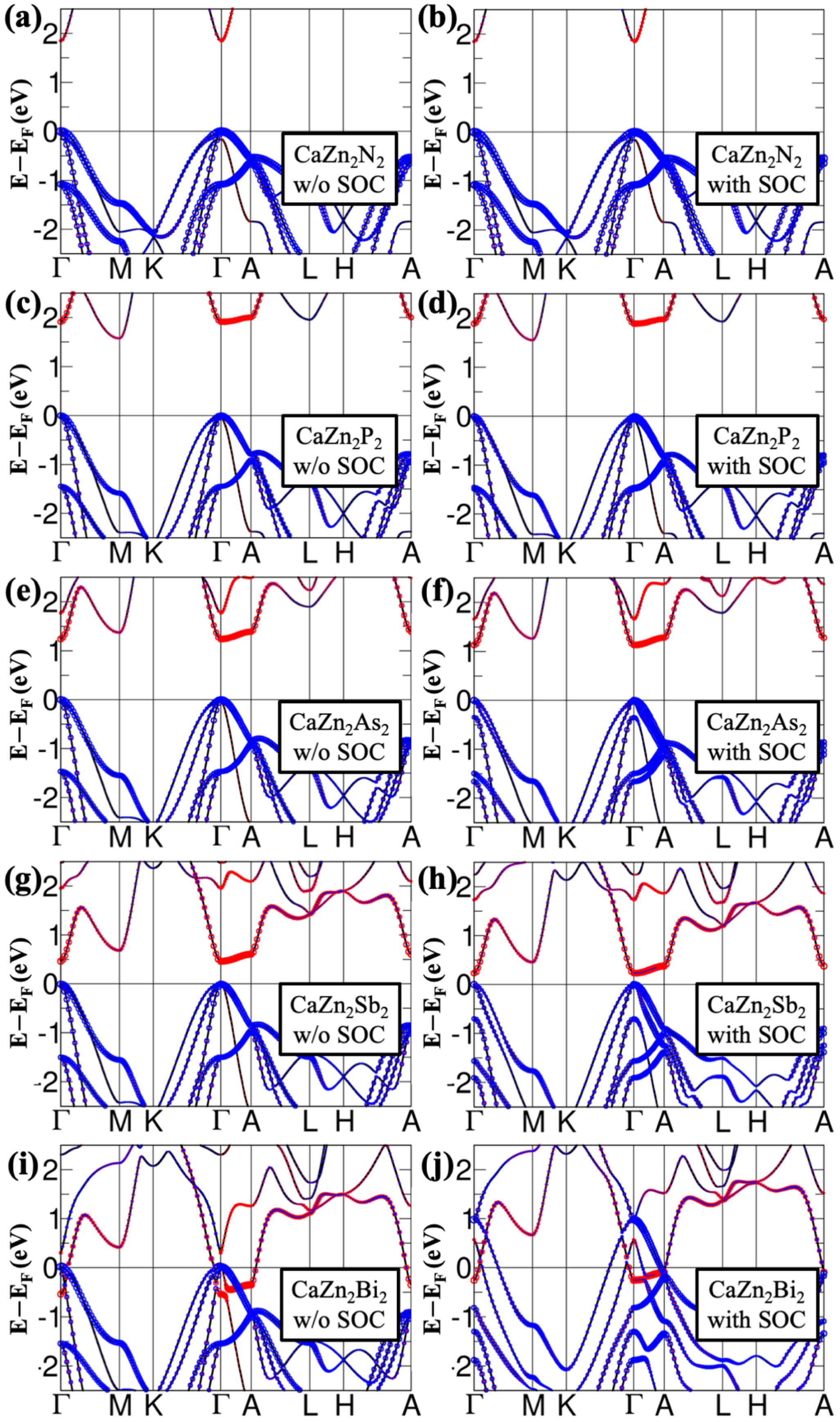

Figure S8. The band structures of bulk $CaCd_2X_2$ (X = N, P, As, Sb, or Bi) under HSE06 (a, c, e, g, and i) without SOC and (b, d, f, h, and j) with SOC in the $CaAl_2Si_2$-type structure. The circle corresponds to the orbital contribution from Cd *s* orbital and Bi p_x and p_y orbitals indicated by red and blue, respectively.

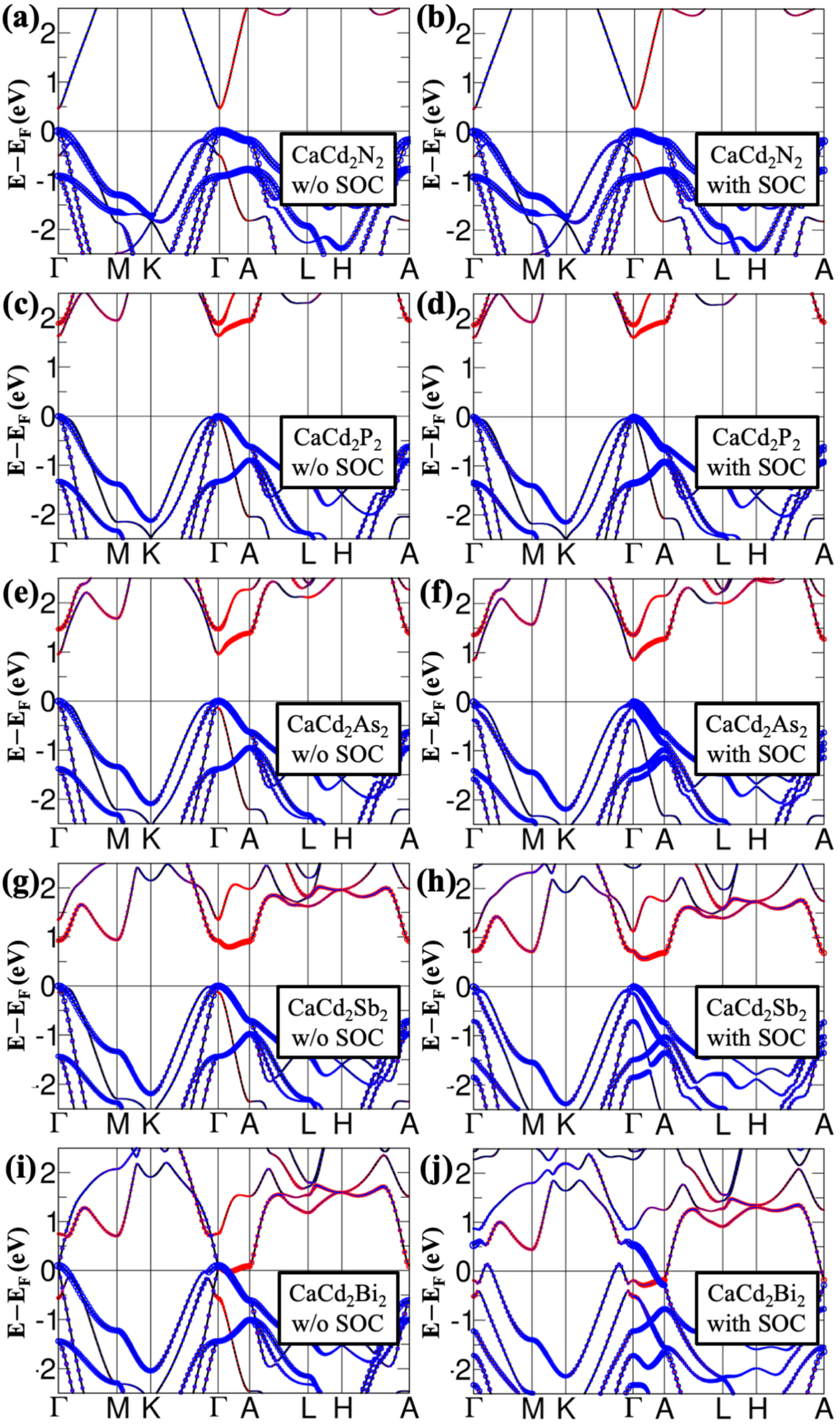

Figure S9. The band structures of bulk $CaCd_2X_2$ (X = N, P, As, Sb, or Bi) under HSE06 (a, c, e, g, and i) without SOC and (b, d, f, h, and j) with SOC in the $CaAl_2Si_2$-type structure. The circle corresponds to the orbital contribution from Cd *s* orbital and Bi p_x and p_y orbitals indicated by red and blue, respectively.

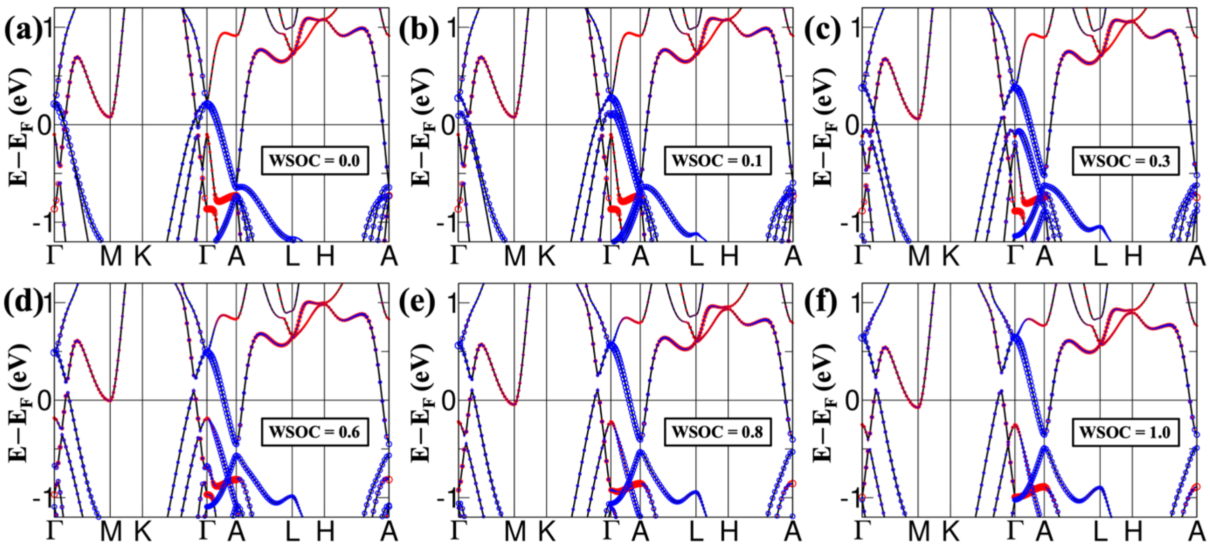

Figure S10. Varying the SOC strength (WSOC) of the bulk $CaZn_2Bi_2$ under GGA-PBE in $CaAl_2Si_2$-type structure. (a) WSOC=0.0, (b) WSOC=0.1, (c) WSOC=0.3, (d) WSOC=0.6, (e) WSOC=0.8, and (f) WSOC=1.0. The circle corresponds to the orbital contribution from Cd *s* orbital and Bi p_x and p_y orbitals indicated by red and blue, respectively.

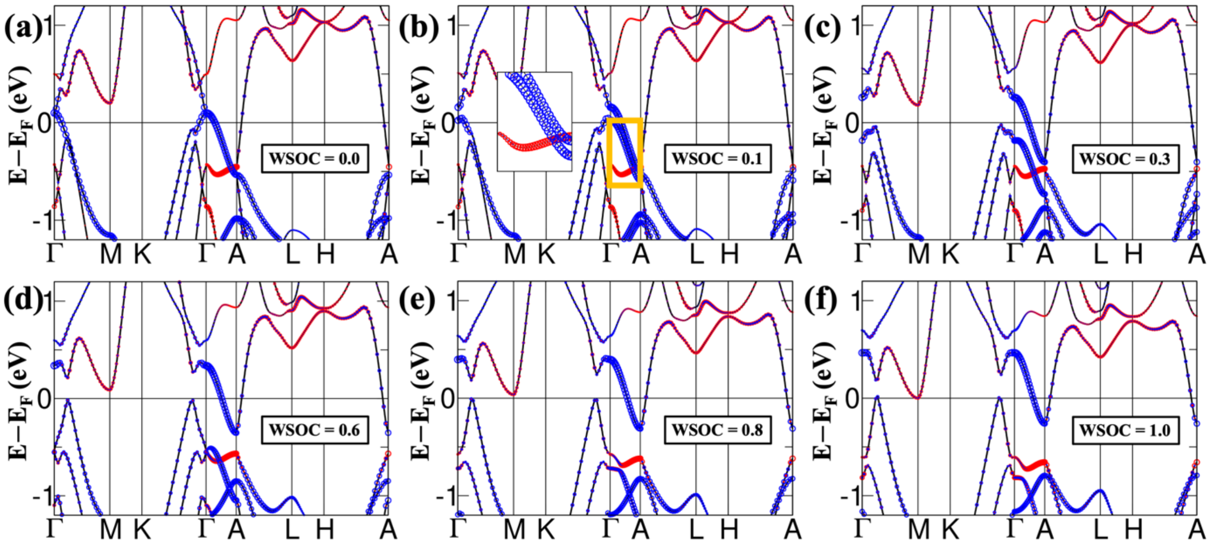

Figure S11. Varying the SOC strength (WSOC) of the bulk $CaCd_2Bi_2$ under GGA-PBE in $CaAl_2Si_2$-type structure. (a) WSOC=0.0, (b) WSOC=0.1, (c) WSOC=0.3, (d) WSOC=0.6, (e) WSOC=0.8, and (f) WSOC=1.0. The circle corresponds to the orbital contribution from Cd *s* orbital and Bi p_x and p_y orbitals indicated by red and blue, respectively. The inset highlighted by the orange square shows the band splitting along the Γ to A direction.

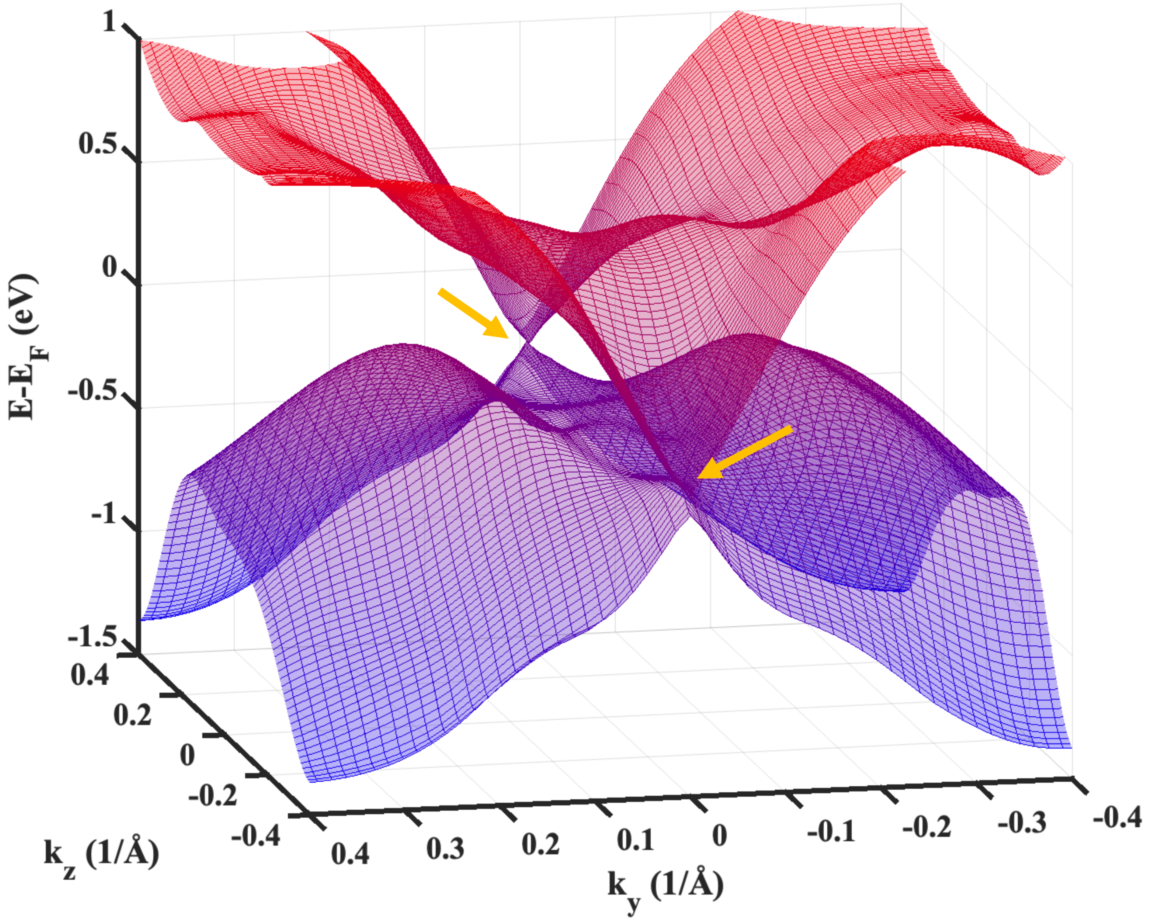

Figure S12. 3D band structure of $CaCd_2Bi_2$ with SOC under HSE06 in $CaAl_2Si_2$-type structure. The Dirac cones are highlighted with orange arrows.
